# Prognostic Evaluation of Patients with Rectal Neuroendocrine Neoplasms and Hepatic Metastases: A SEER Database Analysis

**DOI:** 10.1155/2022/2451282

**Published:** 2022-03-26

**Authors:** Lijun Yan, Yanling Xu, Jialing Pan, Jian'an Bai, Qin Long, Na He, Ping Hu, Min Liu, Hanzhen Ji, Xiaolin Li, Qiyun Tang

**Affiliations:** ^1^Department of Gerontology, The First Affiliated Hospital of Nanjing Medical University, 300 Guangzhou Rd, Nanjing, 210029 Jiangsu Province, China; ^2^Department of Hepatology, Nantong Third People's Hospital Affiliated to Nantong University, 60 Qingnian Rd, Nantong, 226000 Jiangsu Province, China; ^3^Department of Library, Nantong Third People's Hospital Affiliated to Nantong University, 60 Qingnian Rd, Nantong, 226000 Jiangsu Province, China

## Abstract

**Background:**

This study is aimed at investigating the clinical characteristics and prognosis-affecting factors of patients with rectal neuroendocrine neoplasms (r-NENs) and hepatic metastases and offering a scientific-theoretical basis for selective use of an optimized treatment method for r-NENs.

**Methods:**

This study was retrospectively evaluated based on the analysis of the data from Surveillance, Epidemiology, and End Results (SEER) database between 2010 and 2016.

**Results:**

A total of 4,723 r-NEN patients were enrolled in this study, including 168 patients with hepatic metastases (3.56%). Kaplan-Meier analysis revealed that the overall survival (OS) of patients with hepatic metastases receiving primary tumor excision was obviously greater than that of patients without receiving primary tumor excision (OS: nonsurgical patients vs. patients undergoing local resection: *P* < 0.0001 and nonsurgical patients vs. patients undergoing radical resection: *P* < 0.0001); the patients with hepatic metastases in the chemotherapy group had a significantly worse prognosis compared with those in the nonchemotherapy group (OS: *P* = 0.021). Multivariate cox regression analysis revealed that independent affecting factors of overall and tumor-related prognoses in patients with hepatic metastases included tumor grade (G3 and G4), surgical treatment, and chemotherapy.

**Conclusion:**

Among r-NEN patients with hepatic metastases, those undergoing radical excision of lower-grade tumors and chemotherapy will have a better prognosis.

## 1. Background

Neuroendocrine neoplasms (NENs) are originated from the neuroendocrine cells in the organs differentiating from the digestive tract primordia (anterior, middle, and posterior intestine) during the embryonic period. As a highly heterogeneous malignancy, formerly known as carcinoid tumor, NENs can occur in many parts of the body, especially in the digestive and respiratory systems [1, 2]. According to the statistical data from the SEER database using the SEER∗Stat 8.3.5 software, the incidence rate of NENs showed an upward trend year by year, which reached up to 6.98/100,000 in 2012 [3]. With the popularization and mature application of endoscope and related immunohistochemical techniques, the detection rate and diagnostic rate of r-NENs have increased year by year. The incidence rate of r-NENs in 2012 was 1.04/100,000, which had increased by 8 times in the past 40 years. The rectum has become the most common pathogenic site of NENs in posterior intestine [3].

Most r-NENs are nonfunctional, with a more insidious onset and a relatively good prognosis. However, the rectal adenocarcinoma and r-NENs have malignant biological behaviors such as lymph node metastases and distant metastases. The incidence of distant metastases of r-NENs is 3.7% (0.4-13.0%), and the main metastatic organ is hepatic, which is speculated to be related to the immune microenvironment of the liver by some researchers [4]. Surgery is currently the preferred method for the treatment of primary tumors and metastatic tumors of r-NENs [5, 6], and the interventional treatments (e.g., hepatic artery embolization chemotherapy, radiofrequency ablation, and external beam radiotherapy) can be considered when it is impossible to carry out surgical treatment [7]. The chemotherapy and molecular targeted therapy for r-NENs mostly refer to the medication regimen of pancreatic neuroendocrine neoplasms (p-NENs), which can make patients achieve partial remission or disease stability, thus prolonging the overall survival (OS) of patients [8–10]. Base on the SEER database, there were several studies on r-NENs. However, there are few studies on r-NENs with hepatic metastases. In this study, the patients with definite pathological diagnosis of r-NENs were selected from the SEER database, their clinicopathological characteristics were retrospectively analyzed, and special attention was paid to the risk factors of hepatic metastases in patients with r-NENs, and the prognoses of patients with r-NENs and hepatic metastases undergoing different treatments and relative affecting factors were evaluated.

## 2. Material and Methods

### 2.1. Data Sources

The data used in this study were selected from the SEER database, which were newly published in November 2019. Since the detailed information about distant metastases in tumor patients before 2010 was not recorded into the SEER database, the patients pathologically diagnosed with r-NENs from 2010 to 2016 were selected and analyzed. Before the study began, an agreement on data sharing was achieved from the owner of the SEER database.

### 2.2. Study Subjects

**Figure 1:**
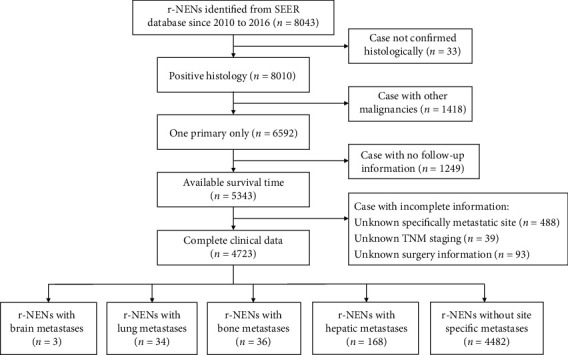
Flow chart of case screening.

The data of patients pathologically diagnosed with r-NENs between 2010 and 2016 were extracted from the SEER database and analyzed by the SEER∗Stat 8.3.5 software. Inclusion criteria are as follows: tumor site-specific codes in the SEER database: C19.9 (rectosigmoid) and C20.9 (rectum) and International Classification of Diseases for Oncology, Third Edition (ICD-O-3) histology codes: 8013/3 (large cell neuroendocrine carcinoma), 8041/3 (small cell carcinoma), 8240/3 (carcinoid tumor), 8244/3 (composite carcinoid), 8246/3 (neuroendocrine carcinoma), and 8249/3 (atypical carcinoid tumor). Exclusion criteria are as follows: (1) the patients had multiple primary tumors; (2) the patients had unknown follow-up information; (3) the patients had unknown distant metastases; (4) the patients did not undergo surgical treatment or had unknown surgical treatment; (5) the patients had unknown tumor grade and T stage. As shown in Figure 1, a total of 4,723 patients with complete data on pathological and clinical characteristics and follow-up were enrolled, including 168 patients with hepatic metastases, 34 patients with lung metastases, 36 patients with bone metastases, and 3 patients with brain metastases.

### 2.3. Observation Index

The clinical data (including age, race, gender, tumor grade, tumor stage, tumor size, distant metastatic site, whether the primary tumor was treated by surgery, radiotherapy, and/or chemotherapy) and the follow-up data were collected. The main observational index was the OS of patients. In this study, the tumor survival time was the time interval from the date of diagnosis to death or last follow-up date.

The 8th edition of American Joint Committee on Cancer (AJCC) TNM Staging Standard was used for tumor staging. The collaborative staging was implemented for patients with incomplete AJCC TNM staging. Different from the 2010 WHO Classification of Tumors, the tumors in the SEER database were divided into well differentiated (G1), moderately differentiated (G2), poorly differentiated (G3), and undifferentiated (G4) tumors according to the degree of histological differentiation.

In addition, the data on surgical procedure were derived from “RX Sum-Surg Prim Site (1998+),” and related codes from the SEER database are shown in Supplementary Table 1. The photodynamic therapy, electrocautery, cryosurgery, laser excision, polypectomy, and excisional biopsy, which were performed in patients enrolled in this study, were described as local resection of the tumor. The endoscopic mucosal resection (EMR) or endoscopic submucosal dissection (ESD) was also included in the local resection; partial or total rectum resection and lymphadenectomy were described as radical resection of the tumor.

### 2.4. Statistical Methods

SPSS, version 25.0(IBM, Armonk, NY, USA), was used for statistical analysis. The counting data were expressed as cases and percentages, and Chi-square test was used for comparison between the two groups; logistic regression was used to analyze the factors affecting the occurrence of hepatic metastases; Kaplan-Meier method was used to draw the survival curve of the patients, and Log-rank method was used to compare the survival curve between the two groups. The variables from the univariate analysis with a *P* < 0.1 (Supplementary Table 2) were included in the multivariate cox regression; subsequently, the independent factors affecting the prognosis were analyzed, and finally, the hazard ratio (HR) and 95% confidence interval (CI) were calculated. All tests were bilateral, and *P* < 0.05 was considered statistically significant.

## 3. Results

### 3.1. Clinicopathological Characteristics of r-NENs with Specific Site Metastases

A total of 4,723 patients with r-NENs were enrolled in this study, including 168 patients with hepatic metastases (3.56%). The clinicopathological characteristics of the patients with hepatic metastases are summarized in Table 1. There were significant differences in age, race, tumor grade, T stage, lymph node metastases, and different treatment methods between patients with hepatic metastases and nonhepatic metastases (all *P* < 0.05), but there was no significant difference in gender between two groups (*P* = 0.058).

Among the r-NEN patients with hepatic metastases, 38 patients (22.62%) underwent primary tumor resection, and details of surgical resection of distant metastases were not available in the SEER database. In addition, we also recorded and analyzed the data on whether patients underwent radiotherapy and chemotherapy and found that 34 patients (20.23%) underwent radiotherapy and 114 patients (67.86%) underwent chemotherapy.

### 3.2. Affecting Factors of Hepatic Metastases in Patients with r-NENs

As shown in Table 2, the results of this study indicated that age, race, and gender were not the factors affecting the occurrence of hepatic metastases, while tumor grade, tumor size, T stage, and lymph node metastases were the factors affecting the occurrence of hepatic metastases. Higher tumor grade, larger tumor diameter, and later T stage with a presence of lymph node metastases caused greater risk of hepatic metastases.

### 3.3. Survival Analysis of Patients with r-NENs and Hepatic Metastases Treated by Different Methods

Kaplan-Meier survival analysis (Table 3) showed that the 1-, 3-, and 5-year OS rates of patients with hepatic metastases who did not undergo primary tumor resection (hereinafter referred to as “nonsurgical group”) were 29.8%, 10.4%, and 1.5%, respectively; the 1-year, 3-year, and 5-year OS rates of patients undergoing local resection of primary tumor (hereinafter referred to as the “local resection group”) were 84.6%, 76.9%, and 41.2%, respectively; the 1-, 3-, and 5-year OS rates of patients undergoing radical resection of primary tumor (hereinafter referred to as the “radical resection group”) were 72.0%, 43.7%, and 43.7%, respectively. The median OS in the nonsurgical group, the local resection group, and the radical resection group was 8, 47, and 29 months, respectively.

In addition, the 1-, 3-, and 5-year cancer-specific survival (CSS) rates in the nonsurgical group, the local resection group, and the radical resection group were similar to 1-, 3-, and 5-year OS rates. The 1-, 3-, and 5-year CSS rates of patients with hepatic metastases undergoing chemotherapy (hereinafter referred to as the “chemotherapy group”) were 34.3%, 16.8%, and 7.9%, respectively; the 1-, 3-, and 5-year CSS rates of patients with hepatic metastases not undergoing chemotherapy (hereinafter referred to as the “nonchemotherapy group”) were 60.4%, 36.1%, and 23.7%, respectively. The median CSS rates in the chemotherapy group and the nonchemotherapy group were 9 and 24 months, respectively. Additionally, the 1-, 3-, and 5-year CSS rates of patients with hepatic metastases undergoing radiotherapy (hereinafter referred to as the “radiotherapy group”) were 38.2%, 16.3%, and 16.3%, respectively; the 1-, 3-, and 5-year CSS rates of patients with hepatic metastases not undergoing radiotherapy (hereinafter referred to as the “nonradiotherapy group”) were 43.7%, 24.2%, and 12.0%, respectively. The median OS in both the radiotherapy group and the nonradiotherapy group was 10 months.

**Figure 2:**
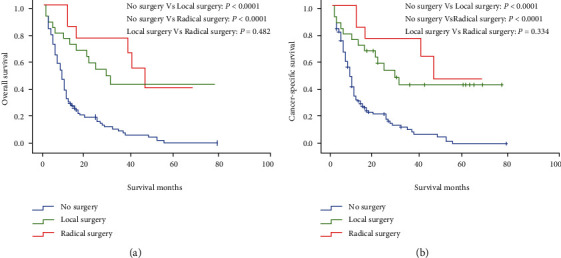
Analysis of the prognosis of patients with r-NENs and hepatic metastases after surgical resection of the primary tumor resection: (a) overall survival and (b) cancer specific survival.

**Figure 3:**
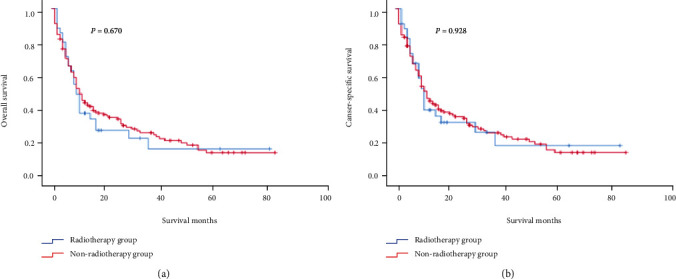
Analysis of the prognosis of patients with r-NENs and hepatic metastases who underwent radiotherapy: (a) overall survival and (b) cancer specific survival.

**Figure 4:**
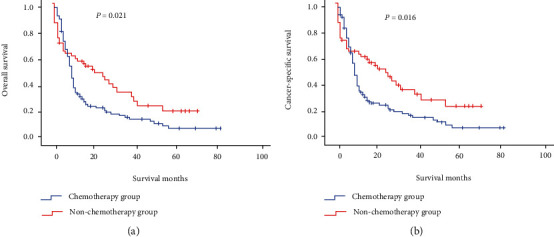
Analysis of the prognosis of patients with r-NENs and hepatic metastases who underwent chemotherapy: (a) overall survival and (b) cancer-specific survival.

As shown in Figure 2, the prognosis of patients with hepatic metastases who underwent surgical resection of the primary tumor was better than that of patients who did not undergo surgical resection of the primary tumor, and the prognosis of patients in the radical resection group was better than that in the local resection group. As shown in Figure 3, there was no statistically significant difference in prognosis of patients with hepatic metastases between the radiotherapy group and the nonradiotherapy group (OS: *P* = 0.670; CSS: *P* = 0.928). As shown in Figure 4, the patients with hepatic metastases in the chemotherapy group had a poorer prognosis compared with those in the nonchemotherapy group (OS: *P* = 0.021; CSS: *P* = 0.016).

### 3.4. Factors Affecting the Prognosis of Patients with r-NENs and Hepatic Metastases

As shown in Table 4, multivariate regression analysis revealed that tumor grade (G3 and G4), surgical treatment, and chemotherapy were independent affecting factors for the overall prognosis and tumor-related prognosis of patients with hepatic metastases, and the patients with higher tumor grade or without primary tumor resection and chemotherapy had a poorer overall prognosis.

## 4. Discussion

r-NENs are difficult to diagnose due to their occult onset and nonspecific clinical manifestations. Therefore, clinicians should attach great importance to it. Some patients present with lower abdominal discomfort (abdominal distension and abdominal pain), perianal discomfort (anal prolapse, feeling of anal falling and distension), change of defecation habits (constipation and diarrhea), and bloody stool, but most of them were generally detected during colonoscopy. With the clinical popularization and mature application of colonoscopy, endoscopic ultrasonography (EUS), and related pathological immunohistochemical techniques, the detection rate and diagnosis rate of r-NENs have increased year by year. The detection rate of patients with r−NENs < 1 cm is 93.3%-100% [11].

It is considered that NENs are low-grade malignant tumors. After lymph node or distant metastasis, the prognosis of NENs is as poor as adenocarcinoma, except for those of low- and medium-grade (G1 and G2) tumors [12]. For NENs with a diameter of less than 1 cm, the probability of metastases is as low as 3-9.7% [13]. The researchers such as Kasuga et al. believed that vascular invasion is the only risk factor for metastases of small r-NENs [6]. Both ENETS (European Neuroendocrine Tumor Society) and NANETS (North American Neuroendocrine Tumor Society) guidelines recommend endoscopic resection of NENs less than 1 cm and without muscular invasion [14, 15]. For NENs with a diameter > 2 cm, the probability of metastases is as high as 56.7-73%, and surgical resection and preventive lymph node dissection are generally recommended. For patients with confirmed metastatic r-NENs, ENETS guideline suggests that only adjuvant therapy such as chemotherapy should be carried out when the patients have no symptoms such as obstruction or rectal bleeding [14]. The results of Smith et al. [16] support this view, and their results showed that resection of primary tumors of high-grade r-NENs cannot improve the prognosis of patients. However, NCCN (National Comprehensive Cancer Network) guidelines suggest that if the tumors can be completely removed, the primary and metastatic tumors should be completely removed; if the tumors are difficult to remove and the local symptoms are severe, palliative resection can be considered to alleviate the symptoms; if the patients have no symptoms, with low tumor load or carcinoid syndrome, it is considered that the patients are treated with octreotide, and the tumor indicators are observed and regularly reexamined. No matter what treatment measures are taken, patients should undergo CT or MRI reexamination every 3 to 12 months. If the disease progression is detected, local treatment of hepatic (chemotherapy, embolization, etc.) and targeted treatment should be considered [17]. Our study showed that among patients with r-NENs and hepatic metastases, the prognosis of patients undergoing primary tumor resection was better than that of patients not undergoing primary tumor resection. Kaplan-Meier survival curve showed that the prognosis of patients undergoing radical resection was better than that of patients undergoing local resection, but there was no statistically significant difference between two groups. In our study, there are only 168 patients with hepatic metastases, and most of them have not undergone primary tumor resection; the number of patients undergoing primary tumor resection was too small (13 and 25); the analysis of propensity score match (PSM) could not be performed. In order to exclude the influence of confounding factors, we further preformed a multivariate cox analysis and the results also showed that compared with the nonsurgical group, the prognosis in the radical resection group was better, and the difference was a statistically significant. After eliminating single factors one by one, we found that tumor grade and tumor size were the factors affecting the results of multivariate Cox regression analysis of patients undergoing primary tumor resection.

The chemotherapy of r-NENs mostly refers to the medication plan for treatment of pancreatic neuroendocrine tumors, which can make the patients achieve partial remission or stable disease and prolong the OS of patients. Cisplatin combined with etoposide or topotecan is the first-line chemotherapy regimen at present. For the patients who cannot tolerate the above chemotherapy regimen, oxaliplatin (or doxorubicin) and vincristine are also used in clinic [18]. Radiotherapy is generally not used to treat r-NENs. In our study, multivariate Cox regression analysis showed that the effect of chemotherapy on the prognosis was opposite to the result indicated by Kaplan-Meier survival analysis, and the single factor was eliminated one by one, and no factor affecting the results was detected. Therefore, a Chi-square test for comparison of patients with hepatic metastases between the chemotherapy group and nonchemotherapy group was performed, and there were statistically significant differences in age, tumor grade, tumor size, surgery, and radiotherapy between two groups (*P* = 0.006, ≤0.001, 0.037, ≤0.001, and 0.004). After the above factors were excluded, the results of chemotherapy analyzed by multivariate Cox regression analysis were consistent with those analyzed by Kaplan-Meier survival analysis, but there was no statistical significance (HR = 0.815, 95% CI: 0.540-1.230, and *P* = 0.329). Because the influences of confounding factors were excluded during multivariate regression analysis, the results were more reliable.

It has been reported in the literature that the risk factors for metastases of NENs include age > 60 years, tumor diameter > 1 cm, muscular invasion and lymphatic vessel or nerve tissue invasion; tumor diameter > 1 cm and lymphatic vessel invasion are independent risk factors [19]. This study showed that tumor grade, tumor size, T stage, and lymph node metastases are the factors affecting the occurrence of hepatic metastases in r-NENs. Higher tumor grade, larger tumor diameter, and later T stage with the presence of lymph node metastases cause greater risk of hepatic metastases.

Our study also has limitations. First of all, the data of tumor metastases were collected from the SEER database between 2010 and 2016, and r-NENs are a rare disease with a low probability of metastases; thus, the sample size in this study is small, which has an effect on the statistical results. Secondly, there is a lack of clinical data such as tumor load, surgical quality, systematic treatment, and drug treatment in the SEER database, which may affect the results of this study.

## 5. Conclusion

This study retrospectively analyzes the clinicopathological characteristics of patients with r-NENs and hepatic metastases, pays special attention to the risk factors of hepatic metastases in patients with r-NENs, and analyzes the prognoses of those patients treated by different treatment methods and respective affecting factors, which may have a certain guiding significance for the clinical treatment of patients with r-NENs and hepatic metastases.

## Figures and Tables

**Table 1:** Clinicopathological characteristics of patients with r-NENs and hepatic metastases.

Characteristics	Hepatic metastases		Total	*P* value
	Yes	No		
*Age (yr)*				<0.0001
<50	45 (26.8%)	1045 (22.9%)	1090 (23.1%)	
50-69	93 (55.4%)	3147 (69.1%)	3240 (68.6%)	
≥70	30 (17.9%)	363 (8.0%)	393 (8.3%)	
*Race*				0.002
White	110 (65.5%)	2451 (53.8%)	2561 (54.2%)	
Black	33 (19.6%)	1096 (24.1%)	1129 (23.9%)	
Other	25 (14.9%)	748 (16.4%)	773 (16.4%)	
Unknown	0 (0.0%)	260 (5.7%)	260 (5.5%)	
*Gender*				0.058
Male	96 (57.1%)	2263 (49.7%)	2359 (49.9%)	
Female	72 (42.9%)	2292 (50.3%)	2364 (50.1%)	
*Grade*				<0.0001
G1	26 (15.5%)	2133 (46.8%)	2159 (45.7%)	
G2	10 (6.0%)	363 (8.0%)	373 (7.9%)	
G3	62 (36.9%)	66 (1.4%)	128 (2.7%)	
G4	34 (20.2%)	31 (0.7%)	65 (1.4%)	
Unknown	36 (21.4%)	1962 (43.1%)	1998 (42.3%)	
*T stage*				
T1	12 (7.1%)	2689 (59.0%)	2701 (57.2%)	
T2	34 (20.2%)	194 (4.3%)	228 (4.8%)	
T3	49 (29.2%)	65 (1.4%)	114 (2.4%)	
T4	19 (11.3%)	20 (0.4%)	39 (0.8%)	
Tx	54 (32.1%)	1587 (34.8%)	1641 (34.7%)	
*N stage*				<0.0001
N0	55 (32.7%)	4255 (93.4%)	4310 (91.3%)	
N1	88 (52.4%)	106 (2.3%)	194 (4.1%)	
Nx	25 (14.9%)	194 (4.3%)	219 (4.6%)	
*Tumor size*				<0.0001
<1 cm	1 (0.6%)	2420 (53.1%)	2421 (51.3%)	
1-2 cm	17 (10.1%)	405 (8.9%)	422 (8.9%)	
>2 cm	101 (60.1%)	173 (3.8%)	274 (5.8%)	
Unknown	49 (29.2%)	1557 (34.2%)	1606 (34.0%)	
*Surgery of the primary*				<0.0001
No surgery	130 (77.4%)	592 (13.0%)	722 (15.3%)	
Local	13 (7.7%)	3730 (81.9%)	3743 (79.3%)	
Radical	25 (14.9%)	233 (5.1%)	258 (5.5%)	
*Radiotherapy*				<0.0001
Yes	34 (20.2%)	74 (1.6%)	108 (2.3%)	
No	134 (79.8%)	4481 (98.4%)	4615 (97.7%)	
*Chemotherapy*				<0.0001
Yes	114 (67.9%)	95 (2.1%)	209 (4.4%)	
No	54 (32.1%)	4460 (97.9%)	4514 (95.6%)	

**Table 2:** Risk factors for hepatic metastases in patients with r-NENs.

Characteristics	Hepatic metastases		*P* value	Multivariate analysis			
	Yes	No		Odds ratio	95% CI		*P* value
					Lower	Upper	
*Age (yr)*			<0.0001				
<50	45 (26.8%)	1045 (22.9%)		Reference			
50-69	93 (55.4%)	3147 (69.1%)		0.712	0.445	1.138	0.156
≥70	30 (17.9%)	363 (8.0%)		0.880	0.460	1.684	0.699
*Race*			0.002				
White	110 (65.5%)	2451 (53.8%)		Reference			
Black	33 (19.6%)	1096 (24.1%)		1.325	0.802	2.188	0.272
Other	25 (14.9%)	748 (16.4%)		1.358	0.762	2.419	0.299
Unknown	0 (0.0%)	260 (5.7%)		0.000	0.000		0.994
*Gender*			0.058				
Male	96 (57.1%)	2263 (49.7%)		Reference			
Female	72 (42.9%)	2292 (50.3%)		0.729	0.488	1.089	0.123
*Grade*			<0.0001				
G1	26 (15.5%)	2133 (46.8%)		Reference			
G2	10 (6.0%)	363 (8.0%)		0.997	0.433	2.298	0.995
G3	62 (36.9%)	66 (1.4%)		6.347	3.286	12.260	≤0.001
G4	34 (20.2%)	31 (0.7%)		6.634	3.036	14.495	≤0.001
Unknown	36 (21.4%)	1962 (43.1%)		1.191	0.690	2.056	0.529
*Tumor size*			<0.0001				
<1 cm	1 (0.6%)	2420 (53.1%)		Reference			
1-2 cm	17 (10.1%)	405 (8.9%)		58.514	7.580	451.674	≤0.001
>2 cm	101 (60.1%)	173 (3.8%)		83.963	9.859	715.040	≤0.001
Unknown	49 (29.2%)	1557 (34.2%)		19.874	2.292	172.330	0.007
*T stage*			<0.0001				
T1	12 (7.1%)	2689 (59.0%)		Reference			
T2	34 (20.2%)	194 (4.3%)		2.793	1.036	7.526	0.042
T3	49 (29.2%)	65 (1.4%)		4.510	1.673	12.156	0.003
T4	19 (11.3%)	20 (0.4%)		5.933	1.762	19.982	0.004
Tx	54 (32.1%)	1587 (34.8%)		2.635	0.939	7.398	0.066
*N stage*			<0.0001				
N0	55 (32.7%)	4255 (93.4%)		Reference			
N1	88 (52.4%)	106 (2.3%)		4.131	2.411	7.079	≤0.001
Nx	25 (14.9%)	194 (4.3%)		6.140	3.390	11.119	≤0.001

**Table 3:** Survival analysis of patients with r-NENs and hepatic metastases treated by different methods.

Treatment modalities	Overall survival rates (%)			Median OS (months)	Cancer-specific survival rates (%)			Median CSS (months)
	1-year	3-year	5-year		1-year	3-year	5-year	
Surgery of the primary cancer								
Nonsurgical	29.8	10.4	1.5	8	32.1	11.7	1.7	8
Local resection	84.6	76.9	41.2	47	84.6	76.9	48.1	47
Radical resection	72.0	43.7	43.7	29	72.0	43.7	43.7	29
Radiotherapy								
No	41.8	23.2	10.8	10	43.7	24.2	12.0	10
Yes	35.3	13.2	13.2	9	38.2	16.3	16.3	10
Chemotherapy								
No	57.1	34.1	19.9	20	60.4	36.1	23.7	24
Yes	32.8	15.4	7.3	9	34.3	16.8	7.9	9

**Table 4:** Multivariate analysis of OS and CSS in the patients with r-NENs and hepatic metastases.

Characteristics	*P* value	Overall survival			*P* value	Cancer-specific survival		
		Hazard ratio	95% CI			Hazard ratio	95% CI	
			Lower	Upper			Lower	Upper
*Age(yr)*								
<50		Reference				Reference		
50-69	0.089	1.482	0.942	2.331	0.169	1.382	0.872	2.189
≥70	0.161	1.537	0.842	2.806	0.357	1.338	0.720	2.484
*Race*								
White		Reference				Reference		
Black	0.852	0.955	0.590	1.546	0.835	1.053	0.646	1.717
Other	0.003	0.390	0.211	0.722	0.009	0.435	0.234	0.809
*Grade*								
G1		Reference				Reference		
G2	0.361	1.646	0.565	4.796	0.316	1.741	0.588	5.156
G3	≤0.001	6.555	2.885	14.896	≤0.001	6.962	2.992	16.198
G4	0.005	3.416	1.447	8.065	0.008	3.353	1.376	8.169
Unknown	0.027	2.568	1.112	5.930	0.021	2.743	1.163	6.466
*T stage*								
T1		Reference				Reference		
T2	0.288	0.599	0.233	1.543	0.296	0.584	0.214	1.599
T3	0.595	0.774	0.301	1.991	0.625	0.779	0.286	2.122
T4	0.592	1.315	0.482	3.586	0.526	1.407	0.490	4.039
Tx	0.425	0.682	0.266	1.746	0.428	0.667	0.245	1.814
*N stage*								
N0		Reference				Reference		
N1	0.057	1.526	0.987	2.360	0.088	1.477	0.943	2.315
Nx	0.029	1.942	1.069	3.526	0.027	2.010	1.085	3.726
*Surgery of the primary*								
No surgery		Reference				Reference		
Local	0.002	0.224	0.087	0.575	0.002	0.205	0.075	0.564
Radical	0.001	0.292	0.138	0.615	0.002	0.308	0.144	0.659
*Chemotherapy*								
Yes		Reference				Reference		
No	0.022	1.812	1.089	3.016	0.032	1.782	1.050	3.024

## Data Availability

Data for this study were obtained from the Surveillance, Epidemiology, and End Results (SEER) Database (2010–2016) that was released in April 2020, based on the November 2019 submission (https://seer.cancer.gov).

## Supplementary Materials

Supplementary Materials Table S1: the “RX Sum-Surg Prim Site(1998+)” variable and related code in the SEER database. Table S2: univariate analysis of overall survival and cancer specific survival in the patients with r-NENs and hepatic metastases.
