# Supplementary material for: Prognostic Evaluation of Patients with Rectal Neuroendocrine Neoplasms and Hepatic Metastases: A SEER Database Analysis
Source: J Oncol. 2022 Mar 26;2022:2451282. doi: 10.1155/2022/2451282 (PMC8976613; doi:10.1155/2022/2451282)
Supplement: Supplementary Materials — Table S1: the “RX Sum-Surg Prim Site(1998+)” variable and related code in the SEER database. Table S2: univariate analysis of overall survival and cancer specific survival in the patients with r-NENs and hepatic metastases. [file 2451282.f1.docx]

**Supplementary Table1 The "RX Sum-Surg Prim Site(1998+)" variable and related code in SEER database**

| Surgical method | Code |
| --- | --- |
| Local resection | 12: Electrocauterization |
|  | 20: Local tumor resection |
|  | 26: Biopsy resection |
|  | 27: Polypectomy |
|  | 21: Photodynamic therapy combined with any one of surgical methods such as 20, 26 and 27 |
|  | 22: Electrocautery combined with any one of surgical methods such as 20, 26 and 27 |
|  | 23: Cryotherapy combined with any one of surgical methods such as 20, 26 and 27 |
|  | 24: Laser ablation combined with any of surgical methods such as 20, 26 and 27 |
|  | 25: Laser resection |
|  | 28: Curettage and electrocauterization |
| Radical resection | 30: Wedge or segmental resection, partial resection, this included, but was not limited to, anterior resection, Hartmann's surgery, lower anterior resection, transperineal rectosigmoidectomy, and total mesorectal resection |
|  | 40: Coloanal anastomosis |
|  | 50: Total rectum resection, this included, but was not limited to, Miles surgery |
|  | 60: Panproctocolectomy |
|  | 70: Proctectomy or proctocolectomy, resection of continuity with other organs, pelvic organ resection |

**Supplementary Table2** **Univariate analysis of overall survival and cancer specific survival in the patients with r-NENs and hepatic metastases**

| **Features** |  | **Overall survival** | |  |  |  | **Cancer-specific survival** | | |
| --- | --- | --- | --- | --- | --- | --- | --- | --- | --- |
|  | **P value** | **Hazard ratio** | **95%*CI*** | |  | **P value** | **Hazard ratio** | **95%*CI*** | |
|  |  |  | **Lower** | **Upper** |  |  |  | **Lower** | **Upper** |
| **Age(yr)** |  |  |  |  |  |  |  |  |  |
| <50 |  | Reference |  |  |  |  | Reference |  |  |
| 50-69 | 0.734 | 1.074 | 0.712 | 1.621 |  | 0.999 | 1.000 | 0.660 | 1.516 |
| >=70 | 0.019 | 1.843 | 1.105 | 3.072 |  | 0.067 | 1.638 | 0.966 | 2.779 |
| **Race** |  |  |  |  |  |  |  |  |  |
| White |  | Reference |  |  |  |  | Reference |  |  |
| Black | 0.050 | 0.641 | 0.411 | 1.001 |  | 0.122 | 0.702 | 0.448 | 1.100 |
| Other | 0.000 | 0.354 | 0.203 | 0.619 |  | 0.001 | 0.388 | 0.221 | 0.680 |
| **Gender** |  |  |  |  |  |  |  |  |  |
| Male |  | Reference |  |  |  |  | Reference |  |  |
| Female | 0.766 | 1.054 | 0.745 | 1.492 |  | 0.987 | 0.997 | 0.695 | 1.429 |
| **Grade** |  |  |  |  |  |  |  |  |  |
| G1 |  | Reference |  |  |  |  | Reference |  |  |
| G2 | 0.335 | 1.634 | 0.603 | 4.427 |  | 0.258 | 1.795 | 0.651 | 4.951 |
| G3 | 0.000 | 7.330 | 3.763 | 14.280 |  | 0.000 | 7.683 | 3.829 | 15.414 |
| G4 | 0.000 | 4.093 | 2.022 | 8.286 |  | 0.000 | 4.027 | 1.919 | 8.450 |
| Unknown | 0.000 | 4.234 | 2.119 | 8.461 |  | 0.000 | 4.496 | 2.188 | 9.236 |
| **Tumor size** |  |  |  |  |  |  |  |  |  |
| <1cm |  | Reference |  |  |  |  | Reference |  |  |
| 1-2cm | 0.877 | 951.019 | 0.000 | 3.58E+40 |  | 0.883 | 843.563 | 0.000 | 8.91E+41 |
| >2cm | 0.853 | 3477.824 | 0.000 | 1.31E+41 |  | 0.859 | 3518.706 | 0.000 | 3.70E+42 |
| Unknown | 0.845 | 5739.650 | 0.000 | 2.15E+41 |  | 0.850 | 5725.478 | 0.000 | 6.02E+42 |
| **T stage** |  |  |  |  |  |  |  |  |  |
| T1 |  | Reference |  |  |  |  | Reference |  |  |
| T2 | 0.713 | 1.170 | 0.506 | 2.704 |  | 0.625 | 1.251 | 0.510 | 3.071 |
| T3 | 0.328 | 1.498 | 0.667 | 3.366 |  | 0.259 | 1.648 | 0.692 | 3.923 |
| T4 | 0.021 | 2.810 | 1.165 | 6.780 |  | 0.012 | 3.288 | 1.296 | 8.346 |
| Tx | 0.099 | 1.959 | 0.880 | 4.357 |  | 0.086 | 2.123 | 0.900 | 5.008 |
| **N stage** |  |  |  |  |  |  |  |  |  |
| N0 |  | Reference |  |  |  |  | Reference |  |  |
| N1 | 0.023 | 1.579 | 1.064 | 2.345 |  | 0.033 | 1.556 | 1.036 | 2.338 |
| Nx | 0.013 | 1.967 | 1.155 | 3.351 |  | 0.013 | 1.991 | 1.153 | 3.436 |
| **Surgery of the primary** |  |  |  |  |  |  |  |  |  |
| No surgery |  | Reference |  |  |  |  | Reference |  |  |
| Local | 0.000 | 0.209 | 0.091 | 0.479 |  | 0.000 | 0.187 | 0.075 | 0.462 |
| Radical | 0.000 | 0.287 | 0.160 | 0.517 |  | 0.000 | 0.306 | 0.169 | 0.551 |
| **Radiotherapy** |  |  |  |  |  |  |  |  |  |
| Yes |  | Reference |  |  |  |  | Reference |  |  |
| No | 0.680 | 0.915 | 0.599 | 1.397 |  | 0.930 | 0.980 | 0.627 | 1.532 |
| **Chemotherapy** |  |  |  |  |  |  |  |  |  |
| Yes |  | Reference |  |  |  |  | Reference |  |  |
| No | 0.026 | 0.644 | 0.437 | 0.949 |  | 0.020 | 0.620 | 0.414 | 0.927 |
